# Differential effects of dietary protein sources on nitrogen metabolism and ileal microbiota in pigs correlated with amino acid release rates

**DOI:** 10.1186/s40104-026-01461-4

**Published:** 2026-07-11

**Authors:** Wenqiang Wang, Wenxuan Zhao, Long Pan, Kaifan Yu, Jing Wang, Weiyun Zhu

**Affiliations:** 1 Laboratory of Gastrointestinal Microbiology, Jiangsu Key Laboratory of Gastrointestinal Nutrition and Animal Health, National Center for International Research On Animal Gut Nutrition, College of Animal Science and Technology, Nanjing Agricultural University, Nanjing, 210095 China; 2 State Key Laboratory of Meat Quality Control and Cultured Meat Development, Nanjing Agricultural University, Nanjing, 210095 China

**Keywords:** Amino acid release rate, Dietary protein sources, Ileal microbiota, Nitrogen metabolism

## Abstract

**Background:**

Dietary protein source is one of the primary factors influencing host nitrogen metabolism and intestinal microbiota. However, conventional diet formulations primarily focus on overall nutritional levels, overlooking the differences in amino acid release rates among various protein sources. This study investigates how different protein sources affect nitrogen metabolism and gut microbiota in finishing pigs.

**Methods:**

The corn-soybean meal diet served as the control (SBM). Fish meal and rapeseed meal isonitrogenously replaced 50% of soybean meal to formulate FM and RPM diets. Twenty-four finishing pigs (DLY; 53.40 ± 2.53 kg) were housed individually in metabolic cages, randomly assigned to three groups (*n* = 8), and fed for 8 weeks.

**Results:**

Feeding the three diets had no significant effect on the growth performance of finishing pigs. Compared with SBM and FM, RPM diet significantly increased fecal nitrogen, but reduced urinary nitrogen, blood urea nitrogen and blood ammonia levels, reduced free amino acids in the small intestinal chyme and plasma, downregulated expressions of amino acid transporters, and showed slower in vitro amino acid release, collectively indicating less amino acid release into the small intestinal lumen and consequently less amino acids into the circulation. In addition, compared with the SBM and FM groups, RPM exhibited increased ileal microbial diversity, characterized by lower relative abundances of *Streptococcus*, *Staphylococcus* and higher relative abundances of *Lactobacillus*, *Turicibacter*. Additionally, microbial cell amino acids and microbial cell protein concentrations were significantly reduced in the RPM group. Correlation analysis showed that the amino acid release rate was negatively correlated with fecal nitrogen, the relative abundance of *Lactobacillus* and *Turicibacter*, while positively correlated with urinary nitrogen, blood urea nitrogen, the relative abundance of *Streptococcus* and *Staphylococcus*, microbial cell protein, and total microbial cell amino acid content.

**Conclusion:**

Dietary protein sources altered host nitrogen metabolism and restructured the ileal microbiota, with the rate of amino acid release appearing to be a key factor associated with these changes.

**Supplementary Information:**

The online version contains supplementary material available at 10.1186/s40104-026-01461-4.

## Introduction

Proteins from different sources, influenced by their structure, affect their enzymatic hydrolysis in the gastrointestinal tract (GIT), regulate amino acid release, and consequently influence nitrogen utilization [1–3]. Even when nutritional levels are identical, variations in dietary protein sources or nitrogen forms can still result in differences in swine growth performance, revealing the limitations of traditional formulations that focus solely on static nutritional levels [4, 5]. Traditional diet formulations overlook the critical differences in digestion kinetics among various protein sources, particularly amino acid release rates. These differences alter the supply dynamics of nutrients in the gut, thereby affecting absorption and metabolism by both the host and the microbiota [6–8].

The porcine intestinal microbiota constitutes a complex ecosystem, playing a crucial role in the digestion, absorption, and metabolism of nutrients, as well as in the maintenance of intestinal health [9–12]. Diet has become one of the primary factors influencing the gut microbiota [13, 14]. Its regulatory mechanism primarily lies in the selective role of nutrient supply, whereby dietary structure drives changes in the composition and function of microbial communities by promoting the growth of bacteria with metabolic preferences for specific nutrients [15]. Notably, among various nutritional factors, the supply status of nitrogen sources plays a central regulatory role in the "diet-microbe" interaction [16–18]. Therefore, the dynamic supply process of dietary nitrogen nutrients, particularly the amino acid release patterns determined by different protein sources, may be a critical link influencing gut microbial ecology and host metabolism.

However, existing research has predominantly focused on the static effects of different protein-source diets on the host and microbiota, while the dynamic process by which different protein sources regulate host-microbe interactions through differences in digestion kinetics—particularly amino acid release rates—remains unclear. Based on this, we hypothesize that diets with different protein sources synchronously affect the host's nitrogen metabolism pathways and the ileal microbial community ecology through the key kinetic characteristic of amino acid release rate. To test this hypothesis, this study used finishing pigs as a model, assessed the amino acid release of different protein-source diets (soybean meal, fish meal, rapeseed meal) through in vitro digestion, and concurrently conducted in vivo experiments to analyze their differential effects on host nitrogen balance, plasma metabolic parameters, expression of small intestinal amino acid transporters, as well as ileal microbial composition and nitrogen utilization.

## Materials and methods

### Protein sources and experimental diets

Three representative protein sources were formulated into three isonitrogenous and isoenergetic diets. Corn-soybean meal diet was selected as the control group (SBM), fish meal and rapeseed meal were substituted for 50% soybean meal in the control group to form FM and RPM diets, respectively. The diets were supplemented with crystalline essential amino acids to meet amino acid requirements. Dietary nutrient levels meet NRC requirements [19]. Composition and nutrition levels of experimental diets are presented in Table 1. Standard procedures were utilized to determine the dry matter (method 930.15; AOAC, 2006) and crude protein content (method 984.13; AOAC, 2006) in the diets [20]. Calcium (GB/T 6436–2018) [21] and Phosphorus (GB/T 6437–2018) [22] levels were determined using a spectrophotometer, and digestibility of amino acids was calculated by their proportions in the formulation according to GB/T 39235–2020 [23].

**Table 1:** Composition and nutrition level of experimental diets (air- dry basis), %^1^

Items	SBM	FM	RPM
Ingredients			
Corn	72.00	72.00	72.00
Soybean meal	13.70	6.85	6.85
Rapeseed meal	-	-	8.34
Fish meal	-	4.42	-
Soybean oil	0.00	0.00	0.57
Rice paddy	10.24	13.25	7.97
Limestone	0.60	0.50	0.60
Dicalcium phosphate	1.00	0.62	1.00
Sodium chloride	0.30	0.30	0.30
L-Lysine-HCl	0.46	0.42	0.57
DL-Methionine	0.10	0.08	0.07
L-Threonine	0.16	0.15	0.19
L-Tryptophan	0.07	0.08	0.08
L-Valine	0.05	0.03	0.08
L-Isoleucine	0.02	0.00	0.08
1% premix^2^	1.00	1.00	1.00
Cr_2_O_3_	0.30	0.30	0.30
Total	100.00	100.00	100.00
Analyzed values of nutrient levels			
Crude protein	12.80	12.93	12.81
Dry matter	90.34	90.48	90.73
Calcium	0.57	0.59	0.61
Total phosphorus	0.50	0.52	0.52
Calculated values of nutrient levels			
Net energy, MJ/kg	10.61	10.61	10.61
Standardized ideal digestibility of AA			
Lysine	0.87	0.87	0.87
Methionine + Cysteine	0.49	0.49	0.49
Threonine	0.53	0.53	0.53
Tryptophan	0.16	0.16	0.16
Isoleucine	0.47	0.47	0.47
Valine	0.58	0.58	0.58
Leucine	1.04	1.05	0.96

^1^SBM: corn-soybean meal diet. FM and RPM diets were formulated by isonitrogenously replacing 50% of soybean meal protein with fish meal and rapeseed meal, respectively

^2^The premix provided the following nutrients per kg of diet: vitamin A 5,200 IU, vitamin B_1_ 1.75 mg, vitamin B_2_ 4.5 mg, vitamin B_5_ 9 mg, vitamin B_6_ 2.25 mg, vitamin B_12_ 0.18 mg, niacin 45 mg, vitamin C 250 mg, vitamin D_3_ 1,400 IU, vitamin E 15 mg, vitamin K_3_ 1.75 mg, choline chloride 500 mg, folic acid 0.8 mg, biotin 0.03 mg, Fe 118 mg, Cu 100 mg, Mn 36 mg, Zn 62 mg, I 0.45 mg, Se 0.27 mg

### Animal trial, nitrogen balance experiment and sampling

Animal feeding trials were carried out in the Oasis breeding enterprise in Zhenjiang City, Jiangsu Province, China. A total of 24 healthy crossbred barrows (Duroc × Landrace × Yorkshire, approximately 120 days old) were individually housed in metabolism cages maintained at a constant temperature of 27 ± 2 °C. The metabolic cage was equipped with a feeder and a nipple drinker, ensuring that the experimental pigs had ad libitum access to feed and water. Prior to the trial, a 7-d adaptation period was conducted, during which the control diet was provided as the pre-feeding diet. After a 7-d adaptation period, all pigs were fasted overnight and weighed to obtain their initial body weight and were subsequently randomized into one of three experimental groups for a 56-d trial period. Body weights of the pigs were recorded on d 1 and d 56 of the trial. Throughout the experiment, individual feed intake was recorded daily to calculate average daily feed intake (ADFI), average daily gain (ADG), and the feed-to-gain ratio (F/G).

The feces and urine of all the pigs were collected separately and weighed daily for 4 d (d 50–53) for a nitrogen balance analysis. The collected fecal and urine samples were mixed with 100 mL/L sulfuric acid at a 1:10 ratio (acid/sample), thoroughly homogenized, aliquoted into sample tubes, and stored at −20 °C for subsequent analysis.

On d 56, blood samples were collected from all pigs via the jugular vein. The blood samples were centrifuged at 3,000 × *g* for 20 min, and the supernatant was transferred to new centrifuge tubes and was used for further analysis of biochemical parameters. Following blood collection, the pigs were kept under general anesthesia through intravenous injection of sodium pentobarbital solution (40 mg/kg body weight) and euthanized via jugular exsanguination. After the abdomen was exposed, the GIT of each pig was removed immediately, jejunum and ileum tissues, mucosa and digesta were collected and quick-frozen in liquid nitrogen and then transferred to an ultra-low-temperature refrigerator at −80 °C for storage.

### In vitro simulated gastrointestinal digestion and determination of the release of dietary glucose and amino acids

The diets were ground to pass through a 1-mm sieve. Subsequently, three replicate tubes were prepared for each diet sample, with 0.5 g of sample accurately weighed into each tube, for in vitro simulated digestion experiments. The in vitro simulated digestion was conducted based on previously described methods with minor adjustments [24]. The digestion process was conducted in two sequential phases: gastric digestion followed by small intestinal digestion. During the gastric phase, 9 mL of 0.1 mol/L phosphate buffer (pH 3.5) was added to each tube, mixed well, and then the pH was adjusted to 3.5 using either a 1 mol/L HCl or a 1 mol/L NaOH solution. Then 1 mL of a freshly prepared pepsin solution was added, containing 50 mg pepsin (P7000, Sigma-Aldrich, Saint Louis, USA). To eliminate bacterial interference, 0.5 mL chloramphenicol solution (1 mg/mL, C8050, Solarbio, Beijing, China) was added and incubated with shaking at 39 °C for 2 h. At the end of gastric phase, 10 mL of 0.08 mol/L NaOH solution was added to adjust the pH to 6.8. Subsequently, 5 mL simulated intestinal fluid was added to each reaction mixture, followed by a 6-h incubation at 39 °C. The simulated intestinal juice was 0.2 mol/L phosphate buffer at pH 6.8, containing pancreatin (0.14 g/mL; P7545, Sigma-Aldrich, Saint Louis, USA). To monitor the release kinetics of amino acids and glucose, suspension samples were collected at gastric digestion time points (0, 60, and 120 min) and small intestinal digestion time points (0, 30, 60, 90, 120, 240, and 360 min). All collected samples were immediately placed on ice to stop the digestion and then stored in a − 20 °C freezer. Total amino acid content was determined using the micro amino acid content assay kit (BC1575, Solarbio, Beijing, China) and glucose content was determined using a glucose assay kit (F006-1-1, Jiancheng, Nanjing, China).

### Amino acid measurement

The free amino acids (FAAs) in chyme, blood and microbial cell amino acids were determined by o-phthaldialdehyde (OPA) derivatization followed by high-performance liquid chromatography [25]. Analysis was performed using an Agilent 1220 Infinity LC system coupled to a fluorescence detector (Agilent Technologies Inc., Santa Clara, CA, USA). Microbial separation was performed using differential centrifugation [26]. Microbial cell amino acids were quantified after acid hydrolysis with 6 mol/L HCl at 110 °C for 24 h. Then, a 1-mL aliquot was dried under a nitrogen stream, followed by reconstitution in 1 mL of sterile deionized water. The subsequent steps were the same as those of the FAA analysis.

### Gene expression analysis in jejunal and ileal mucosa

Total RNA was isolated using an RNA extraction kit (Aidlab, Beijing, China) based on the TRIzol method, quantified using a Nanodrop 2000 spectrophotometer (Thermo Fisher Scientific, MA, USA), and reverse transcribed into cDNA using the Evo M-MLV RT Kit (Accurate Biology, Hunan, China) according to the manufacturer’s protocol. Gene expression levels were detected by real-time quantitative PCR (qPCR) using SYBR Green reagent, and fluorescence was detected by using an ABI 7300 sequence detector (Applied Biosystems, CA, USA). The specific primers are shown in Table S1. Beta-actin was used as the reference gene, and the expression of target genes was calculated relative to the reference gene with the 2^−ΔΔCt^ method.

### Measurement of short-chain fatty acids (SCFAs), microbial cell protein (MCP)

The concentrations of SCFAs were quantified using gas chromatography as described in our prior research [27]. The concentration of MCP in digesta was determined using the Coomassie Brilliant Blue method [28].

### DNA extraction, 16S rRNA gene amplification, high-throughput sequencing and bioinformatics analysis of sequencing data

Total genomic DNA in digesta of ileum was extracted from 0.3 g of sample using the TGuide S96 Magnetic Soil/Stool DNA Kit (Tiangen Biotech (Beijing) Co., Ltd.) according to the manufacturer’s instructions. The extracted DNA was assessed for quality and quantity by 1.8% agarose gel electrophoresis, while its concentration and purity were measured using a NanoDrop 2000 UV–Vis spectrophotometer (Thermo Scientific, Wilmington, USA). The hypervariable region V3-V4 of the bacterial 16S rRNA gene was amplified with primer pairs 338F: 5'-ACTCCTACGGGAGGCAGCA-3' and 806R: 5'-GGACTACHVGGGTWTCTAAT-3'. PCR products were checked on an agarose gel and purified using the Omega DNA purification kit (Omega Inc., Norcross, GA, USA). The purified PCR products were collected and the paired-end sequencing (2 × 250 bp) was performed on the Illumina Novaseq 6000 platform. Clean reads were then processed for feature classification to output ASVs (amplicon sequence variants) by DADA2, and ASVs with counts less than 2 across all samples were filtered out. Taxonomy annotation of the OTUs was performed based on the Naive Bayes classifier in QIIME2 using the SILVA database (release 138.1) with a confidence threshold of 70%. Alpha diversity was calculated and displayed by the QIIME2 and R software, respectively. Beta diversity was determined to evaluate the degree of similarity of microbial communities from different samples using QIIME. Kruskal–Wallis test was used to compare α-diversity among treatments. Principal coordinates analysis (PCoA) was used to analyze beta diversity based on the weighted Bray–Curtis distance.

### Calculations and statistical analysis

The formula for calculating the release rate of glucose and total amino acids over specific intervals is as follows:

$$K=\left({Dt}_{2}-{Dt}_{1}\right)/\left({t}_{2}-{t}_{1}\right)$$

In this equation, *t* refers to the incubation time (min), *K* represents the release rate of glucose (g/100 g/min), or total amino acids (mmol/kg/min), *Dt* is the total amount of nutrient released at *t* min.

Graphing and statistical analysis were performed using GraphPad Prism (La Jolla, CA, USA) and IBM SPSS Statistics software (version 27.0, IBM, Armonk, NY, USA). If the data were normally distributed and had equal variances, an ANOVA was performed, followed by the Bonferroni post hoc test. The statistical model used was

$${Y}_{ij}=\mu +{T}_{i}+{e}_{ij},$$

where *Y*_*ij*_ is the response variable for the *j*^th^ observation in the *i*^th^ group, *μ* is the overall mean, *T*_*i*_ is the effect of the *i*^th^ treatment group, and *e*_*ij*_ is the random error term. If the data failed to meet these criteria, the Kruskal–Wallis test was employed, followed by the Mann–Whitney test with false-discovery rate (FDR) correction using the Benjamini–Hochberg method (adjusted *P* < 0.05). Correlations between in vitro amino acid release rates (mean values per diet) and in vivo parameters (mean values per diet) were analyzed using Pearson's correlation coefficient. Data were expressed as means and pooled standard error of the mean (SEM), statistical significance was set at *P* < 0.05.

## Results

During the entire experimental period, all pigs maintained good health conditions, with no clinical signs of diarrhea or health impairment observed.

### Growth performance, plasma biochemistry, and nitrogen balance

There were no significant differences in FBW, ADG, ADFI and F/G of finishing pigs fed three different protein source diets (Table 2, *P* > 0.05). In terms of plasma biochemistry, compared to the SBM and FM groups, the RPM group had lower concentration of BUN, BA and GLOB, higher concentration of ALB (Table 3,* P* < 0.05). To further evaluate nitrogen utilization, a balance trial was conducted. Fecal N content was significantly higher in the RPM group than in the SBM and FM groups (Table 4, *P* < 0.05). Conversely, the RPM group had lower urine N content than the SBM and FM groups (Table 4, *P* = 0.079). N retention, N excretion, and apparent N availability did not differ significantly among the three groups (Table 4, *P* > 0.05).

**Table 2:** Growth performance of finishing pigs fed diets with different protein sources^1^

Items	Treatments			SEM	*P* value
	SBM	FM	RPM		
IBW, kg	53.69	52.88	53.63	1.792	0.939
FBW, kg	102.56	100.94	103.88	2.474	0.706
ADG, g/d	763.67	750.98	785.16	29.424	0.714
ADFI, g/d	2,926.70	2,937.93	2,924.18	63.312	0.987
F/G	3.87	3.94	3.74	0.115	0.511

^1^*SEM* Standard error of the mean, *IBW* Initial Body weight, *FBW* Final Body weight, *ADG* Average daily gain, *ADFI* Average daily feed intake, F/G Feed:gain ratio, *n* = 8

**Table 3:** Plasma biochemistry of finishing pigs fed diets with different protein sources

Items^1^	Treatments			SEM	*P* value
	SBM	FM	RPM		
GLU, mmol/L	3.36	2.54	3.28	0.268	0.081
TC, mmol/L	2.63	2.55	2.64	0.102	0.794
TG, mmol/L	1.09	0.83	0.72	0.138	0.241
TP, g/L	65.15	63.48	63.63	1.509	0.690
ALB, g/L	38.12^b^	41.40^a^	41.96^a^	0.788	0.005
GLOB, g/L	27.04^a^	22.08^b^	21.67^b^	1.284	0.013
GPT, U/L	79.05	78.49	76.76	4.726	0.939
GOT, U/L	56.16	47.63	57.20	4.083	0.638
LDH, U/L	458.15	425.15	462.56	29.299	0.869
BUN, mmol/L	3.45^ab^	3.55 ^a^	2.96 ^b^	0.156	0.032
BA, μmol/L	127.40^ab^	135.30 ^a^	120.39^b^	2.989	0.011

^1^*GLU* Glucose, *TC* Total cholesterol, *TG* Triglycerides, *TP* Total protein, *ALB* Albumin, *GLOB* Globulin, *GPT* Alanine aminotransferase, *GOT* Aspartate aminotransferase, *LDH* Lactate dehydrogenase, *BUN* Blood urea nitrogen, *BA* Blood ammonia, *n* = 8

^a,b^ Mean values with different superscript letters were significantly different (*P* < 0.05)

**Table 4:** Nitrogen balance in finishing pigs fed diets with different protein sources

Items^1^	Treatments			SEM	*P* value
	SBM	FM	RPM		
N intake, g/d	55.71	54.70	55.68	3.750	0.977
Fecal N, g/d	4.17^b^	4.28^b^	5.08^a^	0.262	0.047
Urinary N, g/d	8.26	7.86	6.49	0.543	0.079
N excretion, g/d	12.44	12.15	11.58	0.905	0.675
N retention, g/d	43.27	42.55	44.10	3.231	0.944
Apparent N availability, %	77.16	77.43	79.00	1.412	0.617

^1^ N excretion = Fecal N + Urinary N, N retention = N intake – N excretion

Apparent N availability = (N retention/N intake) × 100%, *n* = 8

^a,b^ Mean values with different superscript letters were significantly different (*P* < 0.05)

### Ileal microbial diversity, composition, metabolites and microbial nitrogen

**Fig. 1:**
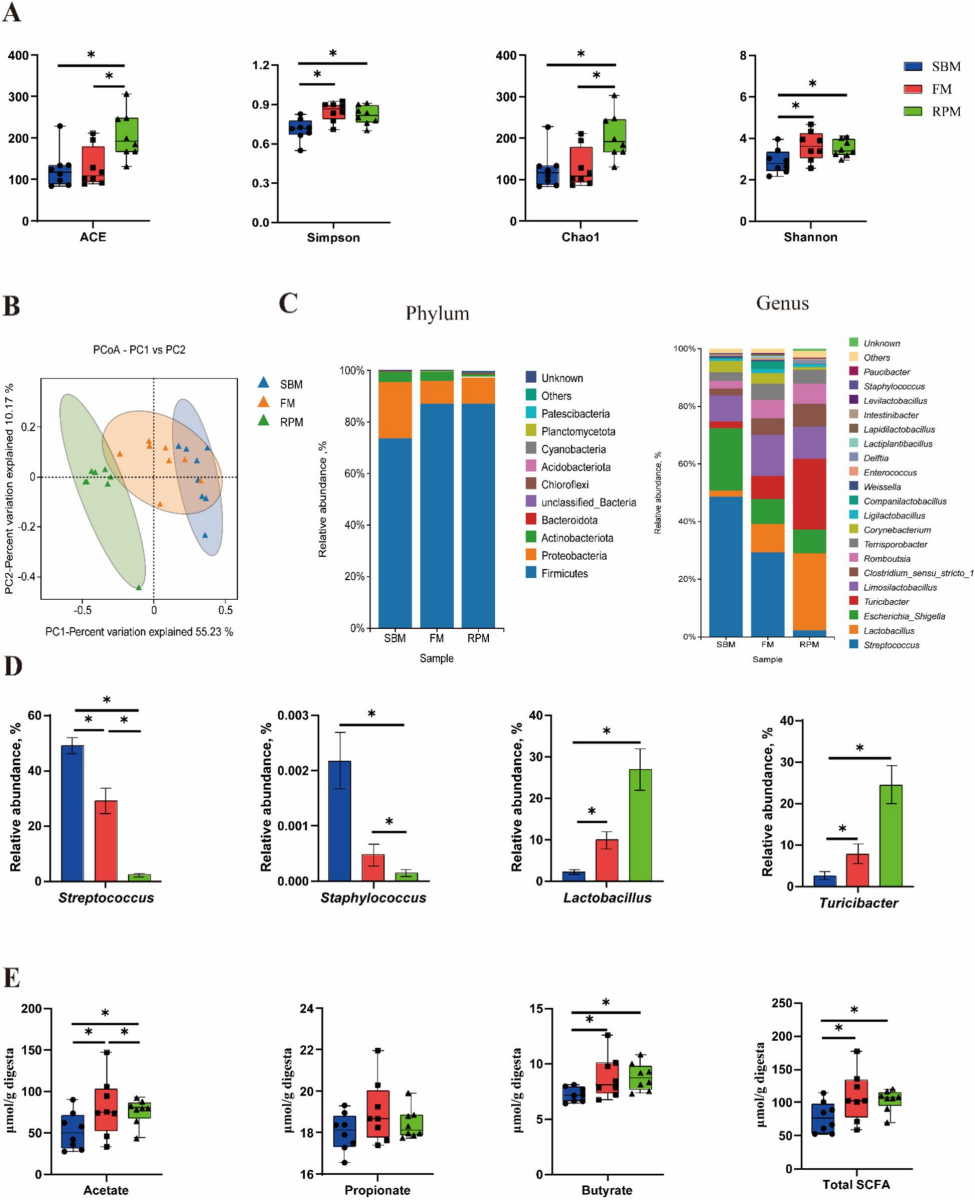
Composition and metabolites of ileal microbiota in pigs fed diets with different protein sources. **A** Alpha diversity. **B** Beta diversity: Principal coordinates analysis (PCoA). **C** Relative abundance of bacteria at phylum and genus level. **D** Ileal microbiota differences at the genus level in response to three dietary protein sources. **E** Ileal acetate, propionate, butyrate, total short-chain fatty acids (Total SCFAs). *n* = 8, ^*^P < 0.05

**Fig. 2:**
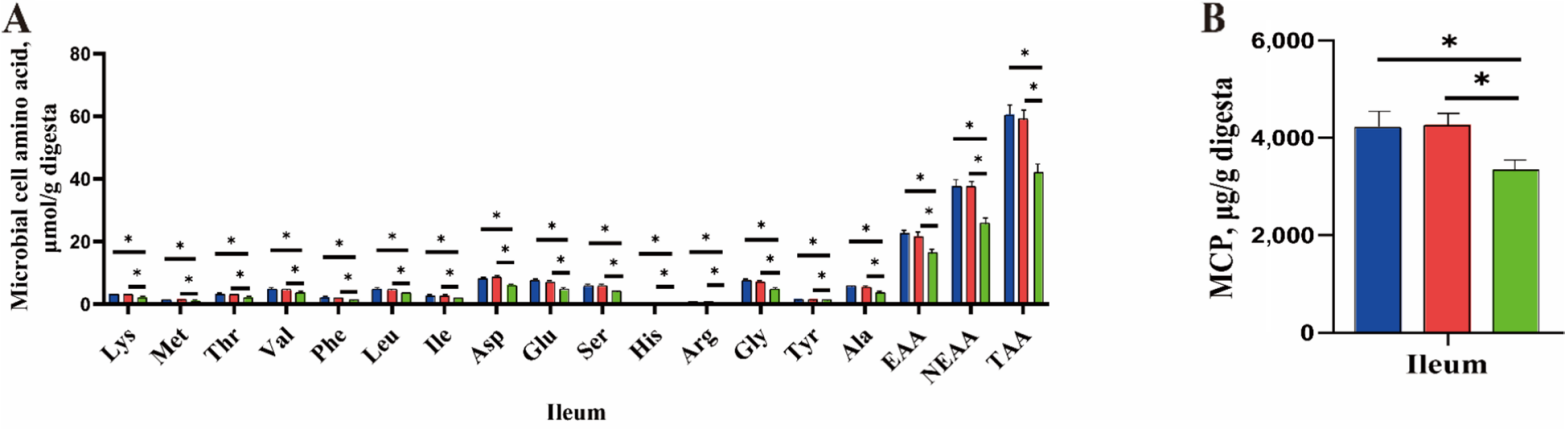
Comparison of ileal microbial nitrogen in response to different protein source diets. **A** Microbial cell amino acid contents. **B** Microbial cell protein (MCP). *n* = 8, ^*^ *P* < 0.05

The results of α-diversity indices showed that the ACE and Chao1 were significantly higher in the RPM group than in the SBM and FM groups. Additionally, the Simpson and Shannon indices were significantly higher in the FM and RPM groups than in the SBM group (Fig. 1A, *P* < 0.05). PCoA revealed a statistically significant divergence in ileal microbial community composition among the three groups (Fig. 1B, *P* < 0.05). Analysis of ileal microbiota composition revealed that Firmicutes and Proteobacteria were the predominant phyla across all groups, constituting the core microbial taxa (Fig. 1C). At the genus level, the microbiota was dominated by *Streptococcus* and *Lactobacillus*, followed by *Escherichia-Shigella* and *Turicibacter* in these treatments (Fig. 1C). The SBM and FM groups demonstrated a significantly higher relative abundance of *Streptococcus* and *Staphylococcus* compared to the RPM group. In contrast, *Lactobacillus* and *Turicibacter* were most prevalent in the RPM group, with intermediate abundances in the FM group and minimal representation in the SBM groups (Fig. 1D, *P* < 0.05). Alterations in microbial communities are consistently accompanied by changes in metabolites. Compared to the SBM group, the FM and RPM groups exhibited significantly higher concentrations of acetate, butyrate and total SCFAs (Fig. 1E, *P* < 0.05). The concentrations of ileal microbial cell amino acids and MCP were significantly higher in the SBM and FM groups than in the RPM group (Fig. 2A and B, *P* < 0.05).

### FAAs in jejunal and ileal digesta and plasma, gene expression of amino acid transporters in jejunal and ileal mucosa, and total nitrogen in cecal digesta

**Fig. 3:**
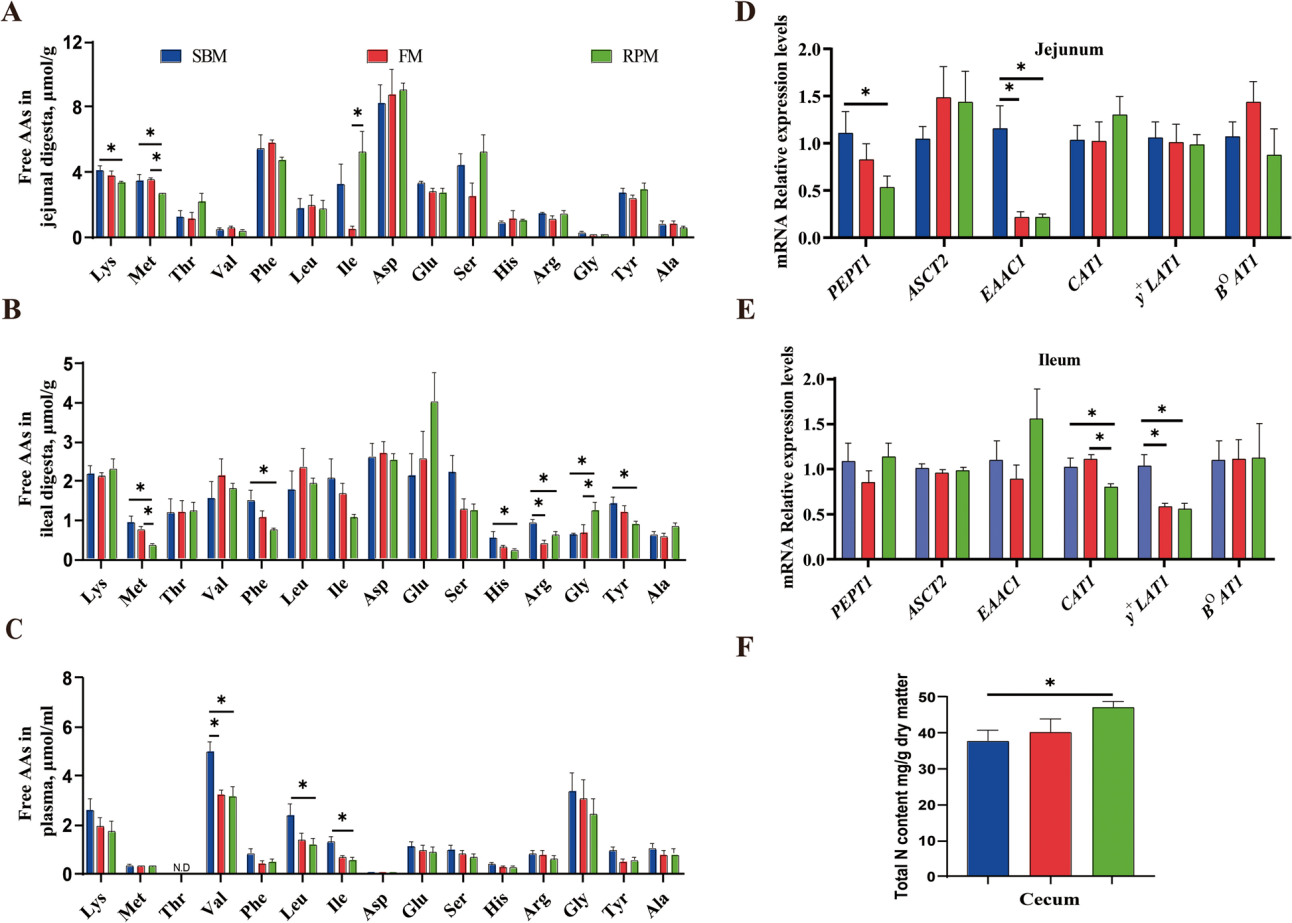
Small intestinal digesta and plasma free amino acid content and expression of amino acid transporter in the small intestinal mucosa of finishing pigs fed diets of different protein sources. **A** Free amino acid content of jejunal digesta. **B** Free amino acid content of ileal digesta. **C** Free amino acid content of plasma. **D** Jejunal amino acid transporter carrier mRNA expression level. **E** Ileal amino acid transporter carrier mRNA expression level. **F** Total nitrogen of cecal digesta. *n* = 8, ^*^P < 0.05

The dietary protein sources significantly modulated the FAA pools in the GIT and systemic circulation. Specifically, compared to the SBM and FM groups, pigs fed the RPM diet exhibited reduced concentrations of Lys and Met in jejunal chyme, lower Met, Phe, His, Arg, and Tyr levels in ileal chyme, lower Val, Leu, and Ile concentrations in plasma (Fig. 3A–C, *P* < 0.05). Amino acid transporters in the small intestinal mucosa are responsible for transporting amino acids from the intestinal lumen to the blood. Compared to the SBM and FM groups, the RPM group exhibited lower mRNA expression of *PEPT1* in the jejunum, lower mRNA expression of *CAT1* in the ileum; compared to the SBM group, the FM and RPM groups showed lower mRNA expression of *EAAC1* in the jejunum, lower mRNA expression of *y*^+^*LAT1* in the ileum (Fig. 3D and E, *P* < 0.05). Total nitrogen of cecal digesta reflects nitrogen consumption in the small intestine. Compared to the SBM and FM groups, the RPM group had higher total nitrogen of cecal digesta (Fig. 3F, *P* < 0.05).

### Release rates of glucose and amino acids during in vitro digestion of diets with different protein sources

**Fig. 4:**
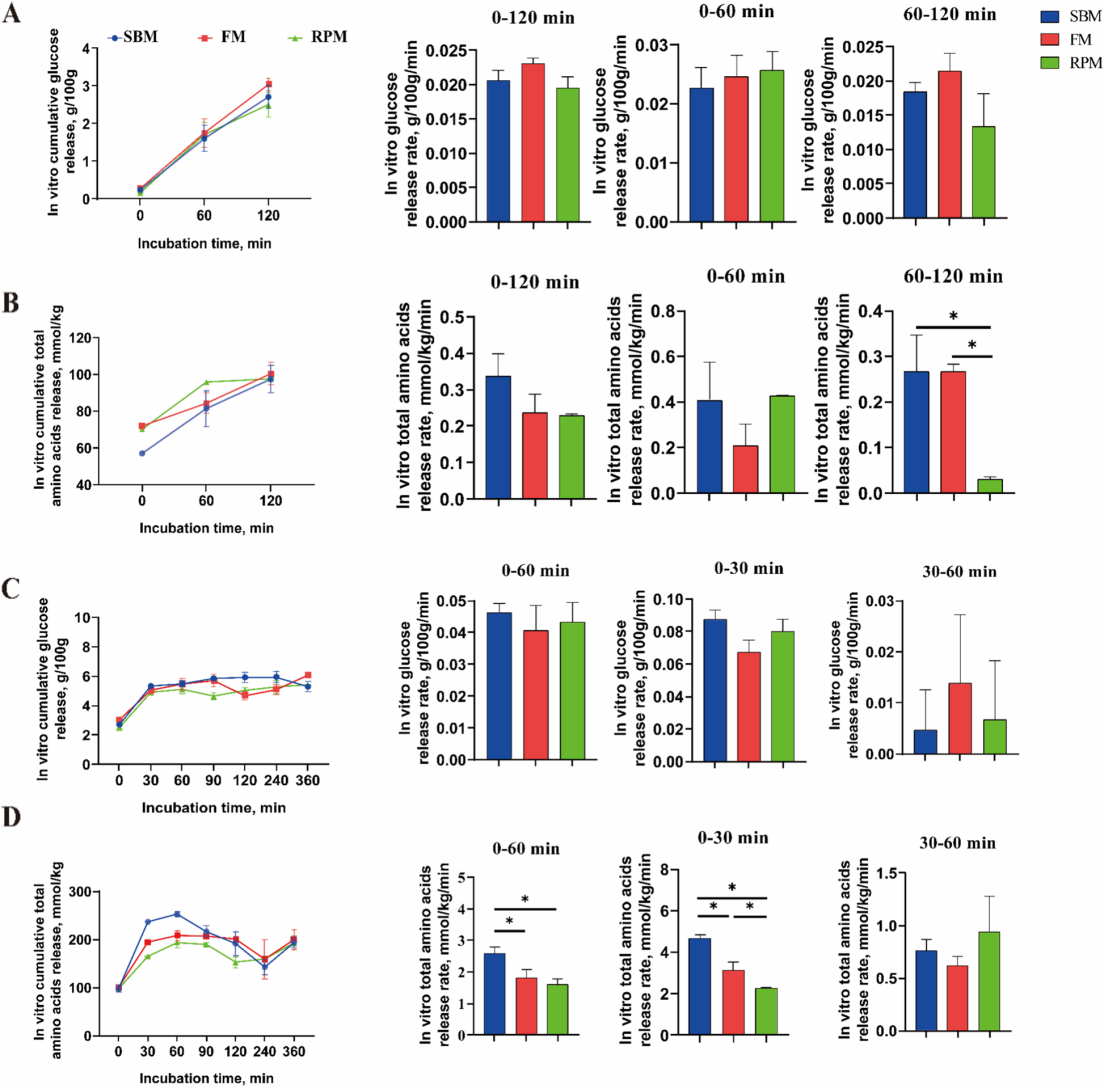
Release of glucose and total amino acids during in vitro digestion of three diets with different protein sources. **A** Cumulative release curve and rate of glucose during gastric digestion. **B** Cumulative release curve and rate of total amino acids during gastric digestion. **C** Cumulative release curve and rate of glucose during small intestinal digestion. **D** Cumulative release curve and rate of total amino acids during small intestinal digestion. *n* = 3, ^*^P < 0.05

To understand the digestion and release of nutrients from different protein-source diets in the GIT of pigs, an in vitro simulated digestion model was employed to mimic gastric and intestinal phases, with monitoring of glucose and amino acid release kinetics. During the gastric digestion phase, dietary glucose release showed no significant differences among the three diets (Fig. 4A), while the total amino acid release rate in SBM and FM diets was significantly higher than in the RPM diet between 60 and 120 min (Fig. 4B, *P* < 0.05). Similarly, during the small intestinal digestion phase, glucose release showed no significant differences among the three diets (Fig. 4C), while the SBM and FM diets exhibited higher total amino acid release rates compared to RPM diet within 0–30 min and 0–60 min (Fig. 4D, *P* < 0.05).

### Correlation analysis between dietary amino acid release rate and host nitrogen metabolism, microbial abundance, and microbial nitrogen utilization

As shown in Table 5, a positive correlation trend was observed between the amino acid release rate and both urinary nitrogen (*r* = 0.897) and BUN (*r* = 0.676) based on group means, while a negative correlation trend was observed with fecal nitrogen (*r* = −0.842). In addition, the amino acid release rate showed a positive correlation trend with the relative abundance of *Streptococcus* (*r* = 0.973) and *Staphylococcus* (*r* = 0.971), MCP (*r* = 0.753), and total microbial cell amino acid content (*r* = 0.824), while a negative correlation trend was observed with the relative abundance of *Lactobacillus* (*r* = −0.934) and *Turicibacter* (*r* = −0.906).

**Table 5:** Correlation analysis between dietary amino acid release rate and host nitrogen metabolism, microbial abundance, and microbial nitrogen utilization^1^

Items	*K* (0–30 min, mmol/kg/min)
Nitrogen metabolism	
Fecal N, g/d	−0.842
Urinary N, g/d	0.897
BUN, mmol/L	0.676
BA, μmol/L	0.333
Microbiota	
Relative abundance of *Streptococcus*, %	0.973
Relative abundance of *Staphylococcus*, %	0.971
Relative abundance of *Lactobacillus*, %	−0.934
Relative abundance of *Turicibacter*, %	−0.906
Microbial nitrogen utilization	
MCP, μg/g digesta	0.753
Total microbial cell amino acid, μmol/g digesta	0.824

^1^The correlation is shown as Pearson *r*, *K* is the rate of amino acid release of diets in vitro, *n* = 3

## Discussion

Based on the inherent properties of different proteins, differences in their amino acid release rates may be one of the key mechanisms leading to variations in nitrogen utilization by the host and gut microbiota. Therefore, the present study systematically evaluated the effects of feeding diets with different protein sources on growth performance, nitrogen metabolism, ileal microbial composition, and microbial nitrogen utilization in finishing pigs. Furthermore, these effects were correlated with the amino acid release rates of the respective diets to elucidate the role of digestion rates in the nutritional regulation of proteins.

The results of the present study indicated that although SBM and FM diets exhibited higher in vitro amino acid release rates, this did not translate into a significant growth performance advantage in finishing pigs. This phenomenon revealed that, under the premise of meeting basic nutritional requirements, the value of dietary protein is determined not only by its static composition but also by the "rhythm" of nutrient release within the digestive tract. Rapidly released amino acids (SBM/FM) did not effectively promote growth but instead drove a fundamental restructuring of nitrogen metabolism pathways. Combined with the findings from the nitrogen balance trial, both the SBM and FM groups showed reduced fecal nitrogen excretion but increased urinary nitrogen output, suggesting that dietary nitrogen from different protein sources is differentially partitioned between fecal and urinary pathways. Animals metabolize excess amino acids through deamination in the liver, which releases them into the bloodstream. Therefore, BUN serves as an indicator of protein intake and amino acid utilization efficiency in swine diets [29, 30]. The present study showed that BUN and BA concentrations were significantly elevated in the SBM and FM groups compared to the RPM group. This indicated that feeding SBM and FM diets results in more amino acids undergoing deamination. The content of FAAs in intestinal chyme and the expression of amino acid transporters can reflect the dynamic status within the intestinal lumen [31, 32]. The present study found that the content of FAAs in small intestinal chyme and plasma, as well as the expression of amino acid transporters, were higher in the SBM and FM groups compared to the RPM group. Additionally, the total nitrogen content in cecal chyme was higher in the RPM group than in the SBM and FM groups. These results collectively indicated that feeding SBM and FM diets released more amino acids into the lumen of the small intestine, leading to increased amino acid entry into the bloodstream. However, the host may be unable to utilize them rapidly, resulting in their subsequent oxidative metabolism and excretion via urine. Unlike animal protein sources, plant protein sources contain antinutritional factors that could interfere with the digestion and absorption process in animals, such as inhibiting the activity of proteolytic enzymes, thereby reducing nitrogen utilization [33]. This may partly explain why the RPM diet had higher fecal nitrogen and total nitrogen in cecal digesta as compared to SBM and FM diets. Future studies are needed to further consider these parameters in order to better understand the underlying mechanisms.

Dietary protein sources are one of the key factors influencing the structure of gut microbiota [14, 34]. The present study found that the FM and RPM groups exhibited higher ileal microbial alpha diversity compared to the SBM. Beta diversity analysis revealed significantly distinct ileal microbial structures among the three groups. The relative abundance results showed that the SBM group had higher relative abundance of *Streptococcus *and* Staphylococcus,* lower relative abundance of *Lactobacillus *and* Turicibacter* compared to RPM group. *Streptococcus* is a predominant commensal bacterium in the small intestine, primarily responsible for the degradation and utilization of proteins [35]. *Lactobacillus* in general can utilize a great variety of carbohydrates [36]. Previous studies have shown that a high-amylopectin diet increases the abundance of *Lactobacillus* and decreases the abundance of *Streptococcus* [37]*.* This suggested that the increased availability of nitrogen in the SBM group led to an enrichment of microbes capable of rapid nitrogen utilization, while the microbial community in the RPM group may have shifted toward a greater reliance on carbohydrate metabolism. In addition, *Streptococcus* is recognized as a potential pathogen, whereas *Lactobacillus* is considered a putative probiotic [38–40]*.* Changes in the relative abundance of these two genera in the ileal microbiota could directly influence the gut's immune homeostasis and nutrient metabolism environment. It is important to note that the experimental diets contained varying proportions of rice paddy, which is rich in non-starch polysaccharides and diverse starches. These components could independently influence the intestinal microbiota, particularly by favoring carbohydrate-preferring bacteria such as *Lactobacillus*. Therefore, the observed shifts in microbial communities should not be attributed solely to dietary protein sources. Instead, these shifts likely reflect a combined effect of protein type and the associated variation in dietary carbohydrate composition. Future studies should eliminate the interference of these factors to better differentiate the specific effects of protein sources from the influence of other dietary components. In addition, the selective enrichment of specific microbes by unidentified nutritional factors in dietary protein sources may be another reason for the observed differences, warranting further investigation. All results indicated that the changes in microbial community structure may be attributed to alterations in the nutrient environment within the small intestine.

Alterations in the microbial composition result in changes in the metabolites produced by microbes [41–43]. The differential health effects of dietary proteins from various sources may primarily stem from variations in host digestibility and subsequent utilization by the gut microbiota. SCFAs are a crucial category of gut microbial metabolites, primarily produced through the fermentation of non-digestible carbohydrates by gut microbiota. They play diverse roles in different physiological processes of the host with implications for human health and disease [44, 45]. Acetate and butyrate exhibit multiple physiological functions, including anti-inflammatory effects and intestinal barrier maintenance [46, 47]. The present study indicated that compared to the SBM group, the higher levels of acetate and butyrate in the FM and RPM groups signify a healthier intestinal environment. Reduced intestinal SCFAs serves as a biomarker for multiple diseases [48]. Previous studies have demonstrated that dietary nitrogen sources serve as the primary contributors to microbial cell protein in the upper intestinal tract of normally nourished pigs [49].This demonstrates that dietary nitrogen nutrients are the primary factor affecting microbial nitrogen utilization. The elevated levels of MCP and total microbial amino acids provide further evidence that ileal microbiota in the SBM and FM groups allocated more dietary nitrogen towards the synthesis of their own cellular components. This may be attributed to the increased availability of nitrogen nutrients in the ileum of the SBM and FM groups compared to the RPM group.

The glucose release rate in pig diets primarily depends on the ratio of amylopectin to amylose in carbohydrates [50]. In our experiment, the types and contents of the main carbohydrates in the diets were largely consistent across all groups. Consequently, no significant differences in glucose release rate were observed among the three groups during in vitro simulated digestion. In vitro simulated digestion showed that SBM and FM diets exhibited higher amino acid release rates compared to RPM diet. This phenomenon may be attributed to the similar digestibility characteristics of soybean meal and fish meal, whereas rapeseed meal exhibited relatively lower digestibility. A previous similar study has indicated that in diets composed of casein and corn gluten meal, casein-based diets facilitate a more rapid release rate of amino acids [8]. The rapid release of amino acids in the stomach and small intestine creates a relatively nitrogen-sufficient environment, which may be one of the key factors influencing host nitrogen metabolism and nitrogen utilization by small intestinal microbiota. A methodological limitation should be acknowledged. To achieve isonitrogenous conditions, crystalline amino acids were added to the experimental diets, with the highest level in the RPM diet. In the in vitro digestion model, crystalline amino acids dissolve instantaneously, whereas protein-bound amino acids require gradual enzymatic hydrolysis for release. This discrepancy may have resulted in an overestimation of the initial amino acid release rate for the RPM diet, and the in vitro release kinetics reported here may not fully represent the true digestive behavior of intact proteins. Future studies utilizing enzymatically hydrolyzed proteins or protein sources that do not require crystalline amino acid supplementation are necessary to validate the true role of amino acid release kinetics in vivo.

To further investigate whether the dietary amino acid release rate is correlated with host nitrogen metabolism, ileal microbial composition, and nitrogen utilization, we conducted a correlation analysis. Given the limited sample size of the in vitro digestion and the inability to directly align with the in vivo data, we calculated Pearson's correlation coefficient (*r*) as a descriptive measure to indicate the strength and direction of the linear trend between group means. An *r* value close to 1 or −1 suggests a consistent co-variation among the three data points, without significance testing. The early stage of in vitro digestion (first 20 or 30 min) corresponds to the rapid-release phase, which is the most sensitive period for distinguishing digestive characteristics. Therefore, we selected the release rate of dietary amino acids during the 0–30 min intestinal digestion phase for correlation analysis with other indicators [2]. Total amino acid release rate of diets with different protein sources correlated negatively with fecal nitrogen excretion, but positively with urinary nitrogen and BUN. This indicated that the release rate of nitrogenous nutrients could influence host nitrogen metabolism to a certain extent, altering the metabolic flux of nitrogen across different pathways in the host. In addition, the total amino acid release rate from diets with different protein sources was positively correlated with the relative abundances of *Streptococcus* and *Staphylococcus*, negatively correlated with the relative abundance of *Lactobacillus* and *Turicibacter*. This suggested that the release rate of nitrogenous nutrients could influence microbial abundance to a certain extent, likely due to the sensitivity of microorganisms to nitrogenous nutrients. Certain unidentified nutrients within protein sources may represent another factor contributing to microbial differences, warranting further investigation in future studies. The total amino acid release rate from diets with different protein sources was positively correlated with MCP and the total amino acid content in microbial cells. MCP and the amino acid content of microbial cells could reflect microbial utilization of nitrogenous nutrients. This indicated that the rate of amino acid release in the small intestine can influence nitrogen utilization by small intestinal microbiota.

## Conclusion

This study indicates that diets with different protein sources have different amino acid release rates, which alter the host's nitrogen metabolism and gut microbiota. The RPM diet, which showed a slow amino acid release rate, resulted in higher fecal nitrogen and lower urinary nitrogen, decreased ileal abundance of potential pathogenic bacteria such as *Streptococcus*, and increased abundance of potentially beneficial bacteria such as *Lactobacillus* as compared with the SBM diet. Correlation analysis showed that the dietary amino acid release rate was negatively correlated with fecal nitrogen, positively correlated with urinary nitrogen, positively correlated with the abundance of *Streptococcus*, and negatively correlated with the abundance of *Lactobacillus*, suggesting an important contribution of dietary amino acid release rate to protein nutrition. These findings provide a theoretical basis for the rational utilization of protein sources to improve nitrogen utilization and gut microecology.

## Supplementary Information

Additional file 1: Table S1. Primers used for the host gene analyses.

## Data Availability

The data analyzed during the current study are available from the corresponding author on reasonable request.

## Abbreviations

ADFI: Average daily feed intake
ADG: Average daily gain
ALB: Albumin
BA: Blood ammonia
BUN: Blood urea nitrogen
BW: Body weight
F/G: Feed gain ratio
FAAs: Free amino acids
GLOB: Globulin
GLU: Glucose
GIT: Gastrointestinal tract
GOT: Aspartate aminotransferase
GPT: Alanine aminotransferase
LDH: Lactate dehydrogenase
MCP: Microbial cell protein
OTU: Operational taxonomic unit
TC: Total cholesterol
TG: Triglycerides
TP: Total protein
SCFAs: Short-chain fatty acids
