# Supplementary material for: Differential effects of dietary protein sources on nitrogen metabolism and ileal microbiota in pigs correlated with amino acid release rates
Source: J Anim Sci Biotechnol. 2026 Jul 11;17:145. doi: 10.1186/s40104-026-01461-4 (PMC13355349; doi:10.1186/s40104-026-01461-4)
Supplement: Supplementary file 1 — Additional file 1: Table S1. Primers used for the host gene analyses. [file 40104_2026_1461_MOESM1_ESM.docx]

**Table S1** Primers used for the host gene analyses

| **Items** | **Primer sequences (5'→3')** | **References** |
| --- | --- | --- |
| PEPT1 | F: CATCGCCATACCCTTCTG | [1] |
|  | R: TTCCCATCCATCGTGACATT |  |
| ASCT2 | F: CAAACGAAGAGCGGAGGAG | [1] |
|  | R: CCTTTCCAAGACGCTGACG |  |
| EAAC1 | F: GGCACCGCACTCTACGAAGCA | [1] |
|  | R: GCCCACGGCACTTAGCACGA |  |
| CAT1 | F: TGCCCATACTTCCCGTCC | [1] |
|  | R: GGTCCAGGTTACCGTCAG |  |
| y^+^LAT1 | F: TTTGTTATGCGGAACTGG | [1] |
|  | R: AAAGGTGATGGCAATGAC |  |
| B^0^AT1 | F: CACAACAACTGCGAGAAGGA | [1] |
|  | R: CCGTTGATAAGCGTCAGGAT |  |

**References**

1. Yu M, Mu C, Yang Y, Zhang C, Su Y, Huang Z, et al. Increases in circulating amino acids with in-feed antibiotics correlated with gene expression of intestinal amino acid transporters in piglets. Amino Acids. 2017;49 (9):1587–99. https://doi.org/10.1007/s00726-017-2451-0.
